# Leiomyosarcoma and Squamous Cell Carcinoma Arising in Mature Cystic Teratoma of the Ovary

**DOI:** 10.1155/2017/7907359

**Published:** 2017-07-02

**Authors:** Tip Pongsuvareeyakul, Kornkanok Sukpan, Somjet Chaicharoen, Surapan Khunamornpong

**Affiliations:** ^1^Department of Pathology, Faculty of Medicine, Chiang Mai University, Chiang Mai 50200, Thailand; ^2^Uttaradit Hospital, Uttaradit, Thailand

## Abstract

The occurrence of malignant transformation in mature cystic teratoma of the ovary is rare, with squamous cell carcinoma being the most common histologic type. Sarcomatous transformation has been rarely described in the literature. We present a case of leiomyosarcoma with a minor component of squamous cell carcinoma arising in mature cystic teratoma of ovary in a 65-year-old woman. The malignant tumor showed two distinct components of sarcomatous and invasive epithelial elements, which were confirmed by immunostaining. To our knowledge, only four cases of leiomyosarcoma in ovarian mature cystic teratoma have been reported and this is a unique case report of leiomyosarcoma and squamous cell carcinoma arising in a mature cystic teratoma of ovary.

## 1. Introduction

Mature cystic teratoma (dermoid cyst) is the most common type of ovarian neoplasms, accounting for approximately 10%–20% of all ovarian tumors [1]. It is a type of germ cell tumor of the ovary and may arise from postmeiotic primordial germ cells [2]. Over 80% of cases are diagnosed in women of reproductive age [3].

Somatic-type cancer (malignant transformation) arising from dermoid cyst is rare and occurs in approximately 1-2% of cases, typically in postmenopausal women [1, 3]. Squamous cell carcinoma is the most common histologic type of malignancy (approximately 80% of cases), followed by adenocarcinoma (approximately 15%) [1, 3]. Other rare types of malignancies include melanoma, carcinoid, thyroid carcinoma, basal cell carcinoma, and sarcoma [3, 4]. Various types of sarcoma arising in an ovarian dermoid cyst have been reported such as angiosarcoma [5], osteosarcoma [6–8], malignant fibrous histiocytoma [9, 10], rhabdomyosarcoma [11], chondrosarcoma [12], and leiomyosarcoma [3, 8, 13, 14].

To our knowledge, only four cases of leiomyosarcoma arising in ovarian dermoid cyst have been described [3, 8, 13, 14]. In one of these cases, an associated component of squamous cell carcinoma in situ was also present. In this report, we present another case of leiomyosarcoma arising in mature cystic teratoma of the ovary, with a coexisting minor component of squamous cell carcinoma.

## 2. Case Presentation

A 65-year-old, gravida 0 para 0, postmenopausal woman presented with abdominal pain for one week. A whole abdominal computed tomography scan showed a large intraabdominal mass (24.2 × 21.2 × 16.9 cm) containing fat, calcification, and soft tissue parts with irregular border, which was suspicious for teratoma of the ovary with malignant component. Multiple peritoneal nodules, intraabdominal lymphadenopathy (aortocaval, perihepatic, and diaphragmatic regions), left adrenal nodule, and two subpleural nodules in the basal parts of both lungs were considered likely metastatic involvement from the ovarian tumor. The serum CA-125 level was 202.5 U/mL (normal < 35 U/mL). The patient underwent an exploratory laparotomy for tumor debulking. Intraoperatively, a 30 cm right ovarian tumor and multiple omental nodules were identified. A total abdominal hysterectomy with bilateral oophorectomy and omentectomy was performed. The suspected metastatic lesions in the upper abdomen including adrenal gland and lungs were not surgically removed.

**Figure 1:**
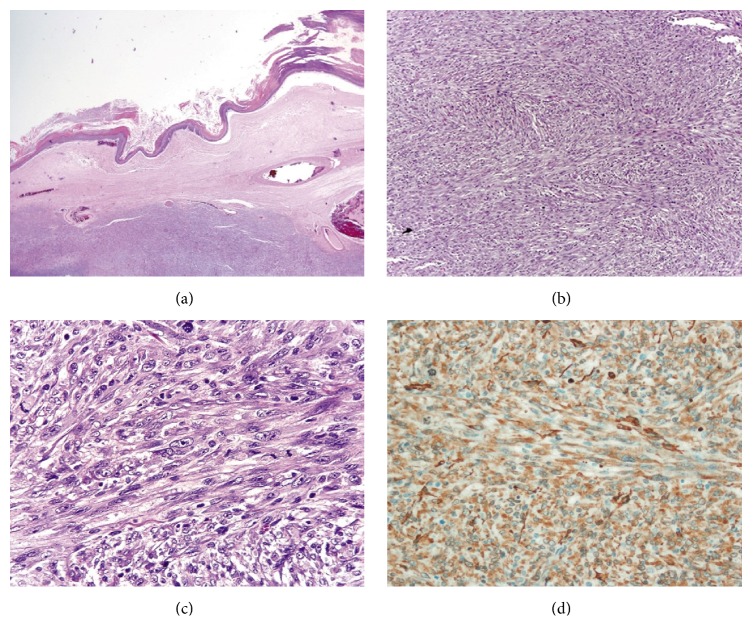
Leiomyosarcoma in mature cystic teratoma of ovary. (a) The cyst lined by keratinized squamous epithelium. Note the intramural sarcomatous component beneath the cyst lining (H&E, 1.25x). (b) Leiomyosarcoma showed intersecting fascicles of malignant spindle cells (H&E, 10x). (c) Leiomyosarcoma exhibited moderate to markedly atypical spindle cells with blunt-ended (or cigar-shaped) nuclei and eosinophilic fibrillar cytoplasm. Note numerous mitotic figures (H&E, 40x). (d) Leiomyosarcoma showed strong and diffuse positivity for smooth muscle actin (IHC stain, 40x).

**Figure 2:**
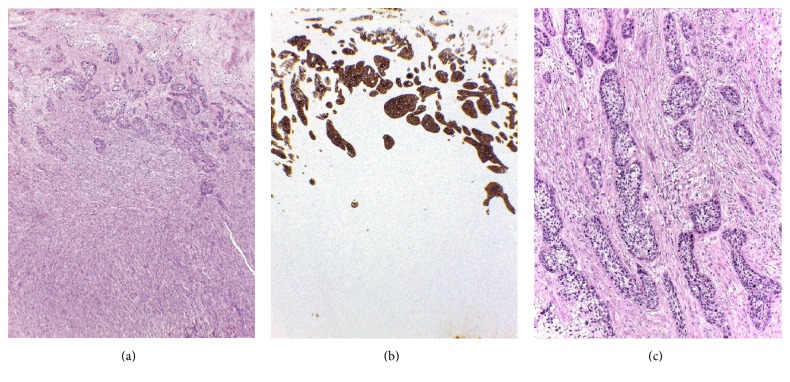
Leiomyosarcoma and squamous cell carcinoma. (a) Squamous cell carcinoma at the upper part of leiomyosarcoma. Note an abrupt demarcation between two components (H&E, 4x). (b) Squamous cell carcinoma was highlighted by positive staining for AE1/AE3, whereas leiomyosarcoma was completely negative (IHC stain, 4x). (c) Infiltrative nests of squamous cell carcinoma surrounded by desmoplastic stromal reaction (H&E, 10x).

Macroscopically, the right ovarian mass was a solid-cystic tumor composed of a 17 cm unilocular cystic part with thick sebaceous material admixed with hairs and a solid mural thickening (13 × 10 × 4 cm) which showed a gray tan trabeculated sectioned surface. The solid part of tumor invaded through the external surface with involvement of the right tube. Microscopically, the cystic part showed a cutaneous lining composed of keratinized squamous epithelium (Figure 1(a)) with associated cutaneous appendages, associated with underneath mature fat tissue and cartilage, typical of a dermoid cyst. The mural solid component was composed of intersecting fascicles of malignant spindle cells with diffuse moderate to marked nuclear atypia. The neoplastic cells showed blunt-ended (cigar-shaped) nuclei with eosinophilic fibrillar cytoplasm (Figures 1(b) and 1(c)). The mitotic count was 13 in 10 high power fields, and atypical mitotic figures were present. Areas of coagulative tumor cell necrosis were noted. The initial set of tissue sampling of 13 histologic sections did not identify any carcinomatous component or dysplastic changes in the dermoid cyst lining. The second-round tissue sampling revealed a 2 cm area of squamous cell carcinoma in the cystic surface overlying the malignant spindle cell component. Nests of squamous cell carcinoma (13 mm in depth) infiltrated into the malignant spindle cell component, but both components were distinctive to each other, without a merging or transitional area of transformation from carcinoma into spindle cells (Figures 2(a) and 2(b)). The infiltrative carcinomatous component was focally surrounded by desmoplastic stroma (Figure 2(c)).

Immunohistochemically, the malignant spindle component showed a diffusely positive reaction for smooth muscle actin (Figure 1(d)) and focal positivity for desmin and muscle-specific actin (HHF-35). The immunostains for h-caldesmon, cytokeratin (AE1/AE3), 34*β*E12, p63, epithelial membrane antigen, calretinin, CD 117, DOG-1, S-100, and HMB-45 were negative. By contrast, the squamous cell carcinoma component was immunoreactive for cytokeratin (AE1/AE3), 34*β*E12, and p63, with negative reaction for h-caldesmon, desmin, and muscle-specific actin (HHF-35). The histologic features and immunoprofile were consistent with a diagnosis of coexisting leiomyosarcoma and squamous cell carcinoma arising in an ovarian dermoid cyst.

The leiomyosarcomatous component infiltrated into right fallopian tube and uterine serosa. Neither myometrial lesion nor intrauterine lesion was identified. The omentum showed multiple metastatic nodules, measuring up to four centimeters in the greatest dimension, consistent with at least FIGO tumor stage IIIC. After the surgery, she had a fever with clinical suspicion of postoperative pneumonia. She developed sepsis with multiorgan failure and died from septic shock one month postoperatively. An autopsy was not performed.

## 3. Discussion

Smooth muscle tumor of the ovary is uncommon, accounting for less than 1% of all ovarian tumors, and it has been proposed to originate from hilar blood vessels, smooth muscle metaplasia of ovarian stromal or theca cells [14]. In the cases with associated ovarian lesion such as endometriosis, teratoma, or mucinous tumor, smooth muscle cells in these lesions may be the origin of ovarian smooth muscle tumors [14]. Leiomyosarcoma of the ovary is a very rare tumor, with approximately 70 cases reported in the English literature [16]. The diagnostic criteria for ovarian leiomyosarcoma are similar to those used for the uterine counterpart [17]. Ovarian leiomyosarcoma typically occurs in postmenopausal women with a mean age of 52.6 years and has an unfavorable prognosis [16]. In the largest reported series of 26 cases of ovarian leiomyosarcoma, a teratomatous component was present in only two cases [14].

In the present case, leiomyosarcoma was the main malignant component associated ovarian dermoid cyst. Only one minor focus of coexisting squamous cell carcinoma was identified after an extensive tissue sampling. The two tumor components showed different histomorphology and immunohistochemical profiles.

The presence of teratomatous background supports an origin of leiomyosarcoma from dermoid cyst. Devouassoux-Shisheboran et al. [18] compared the genetic profiles of malignancy associated with ovarian mature cystic teratoma. The study included eight cases of various types of malignancy including four squamous cell carcinomas, two sarcomas (angiosarcoma and rhabdomyosarcoma), one thyroid carcinoma, and one carcinoid tumor. All malignant components showed a homozygous genotype, which supports a teratomatous nature. Identical genetic profiles between mature teratoma and the malignant components were also detected in seven of eight cases, which is in agreement with the teratomatous origin of malignancy [18].

Regarding leiomyosarcoma, the pattern of immunoexpression of smooth muscle markers may have an implication for the histogenesis [19, 20]. Matsuyama et al. [19] compared immunoexpressions of smooth muscle markers between four leiomyosarcomas of vascular origin and 43 leiomyosarcomas of nonvascular origins. All four vascular leiomyosarcomas showed diffuse positivity for h-caldesmon whereas three of these were only focally positive or negative for desmin. In contrast, 49% of leiomyosarcomas of nonvascular (somatic soft tissue) origin were negative for h-caldesmon, whereas a variable degree of desmin expression was observed in 88% [19]. In the present case, the immunoprofile (desmin-positive/h-caldesmon-negative) may suggest that the leiomyosarcoma component originates from nonvascular smooth muscle cells in teratomatous tissue.

The occurrence of different or mixed types of cancer arising in ovarian dermoid cyst is extremely rare. To our knowledge, only six such cases (including the present case) have been described in the English literature (mean age 66.5 years) [4, 6, 9, 10, 15] (Table 1). In another case reported by Tyagi et al., squamous cell carcinoma in situ in the lining of dermoid cyst coexisted with leiomyosarcoma in a 60-year-old woman. The pathogenesis of the coexistence of different types of malignancy in dermoid cyst has not been clearly elucidated [6, 9]. Possible explanations may include a synchronous biclonal origin (collision tumor) [10] and bidirectional or metaplastic differentiation of a single clone of totipotential cancer cells [4, 6, 9, 15]. An admixture of two malignant components without well-demarcated border is a suggestive evidence for a single clonal origin [9, 15]. In a previous report, Cabibi et al. demonstrated that the sarcomatous component shared similar p53 and p63 immunoexpressions with the squamous cell carcinoma component, whereas the atypical basal cells of the squamous cyst lining expressed aberrant expressions of vimentin, smooth muscle actin, and CD10. Such findings support a single clonal origin or transformation of epithelial component into a mesenchymal component. Epithelial-mesenchymal transformation has been well recognized and this is the explanation for histogenesis of carcinosarcoma (including mixed malignant Mullerian tumor in the female genital tract). In the present case, the absence of transformation area between leiomyosarcoma and squamous cell carcinoma component favors a synchronous biclonal origin (collision tumor). In addition, squamous cell carcinoma was confined to only one minor focus, which is not consistent with a diagnosis of carcinosarcoma.

Prognosis of malignant transformation in mature teratoma depends on the stage of the disease [1, 3]. The majority of patients present with an advanced stage [3, 9] and direct invasion to adjacent organs are the most common route of spread [3]. In cases with disease spreading beyond the ovary (stages II–IV), the long-term survival is worse [9]. The two-year survival for advanced stage is 0%–30% and five-year survival is 0% [9]. The histologic types of tumors other than squamous cell carcinoma (adenocarcinoma or sarcoma) are related to a worse prognosis [3].

## Figures and Tables

**Table 1:** Mixed types of cancer arising in ovarian dermoid cysts.

Authors	Patient age (years)	Tumor size (cm)	FIGO stage	Pathology		Patient outcomes
				Carcinoma	Sarcoma	
Hanada et al. [10]	75	21	N/A	Squamous cell carcinoma	Malignant fibrous histiocytoma (myxoid variant)	NED, for 21 months
Arora and Haldane [4]	78	23	N/A	Adenocarcinoma	No definite differentiation^*∗*^	N/A
Cabibi et al. [15]	69	27	N/A	Squamous cell carcinoma	No definite differentiation^*∗*^	N/A
Allam-Nandyala et al. [6]	55	11.8	N/A	Squamous cell carcinoma	Osteosarcoma	DOD, after 5 months
Savitchi and Rao [9]	58	30	IIIB	Squamous cell carcinoma	Malignant fibrous histiocytoma	DOD, after 5 months
Current case	65	17	At least IIIC	Squamous cell carcinoma	Leiomyosarcoma	DOC, after 1 month

N/A, not available; NED, no evidence of disease; DOD, dead of disease; DOC, dead of unrelated cause. ^*∗*^Focally positive for smooth muscle actin.
